# Regional differences in the validity of self-reported use of health care in Belgium: selection versus reporting bias

**DOI:** 10.1186/s12874-016-0198-z

**Published:** 2016-08-16

**Authors:** J. Van der Heyden, R. Charafeddine, D. De Bacquer, J. Tafforeau, K. Van Herck

**Affiliations:** 1 Department of Public Health and Surveillance, Scientific Institute of Public Health, 14, Juliette Wytsmanstraat, 1050 Brussels, Belgium; 2 Department of Public Health, Ghent University, 185, De Pintelaan, 9000 Ghent, Belgium

**Keywords:** Validity, Use of health care, Health interview survey, Selection bias, Reporting bias

## Abstract

**Background:**

The Health Care Module of the European Health Interview Survey (EHIS) is aimed to obtain comparable information on the use of inpatient and ambulatory care in all EU member states. In this study we assessed the validity of self-reported information on the use of health care, collected through this instrument, in the Belgian Health Interview Survey (BHIS), and explored the impact of selection and reporting bias on the validity of regional differences in health care use observed in the BHIS.

**Methods:**

To assess reporting bias, self-reported BHIS 2008 data were linked with register-based data from the Belgian compulsory health insurance (BCHI). The latter were compared with similar estimates from a random sample of the BCHI to investigate the selection bias. Outcome indicators included the prevalence of a contact with a GP, specialist, dentist and a physiotherapist, as well as inpatient and day patient hospitalisation. The validity of the estimates and the regional differences were explored through measures of agreement and logistic regression analyses.

**Results:**

Validity of self-reported health care use varies by type of health service and is more affected by reporting than by selection bias. Compared to health insurance estimates, self-reported results underestimate the percentage of people with a specialist contact in the past year (50.5 % versus 65.0 %) and a day patient hospitalisation (7.8 % versus 13.9 %). Inversely, survey results overestimated the percentage of people having visited a dentist in the past year: 58.3 % versus 48.6 %. The best concordance was obtained for an inpatient hospitalisation (kappa 0.75). Survey data overestimate the higher prevalence of a contact with a specialist [OR 1.51 (95 % CI 1.33–1.72) for self-report and 1.08 (95 % CI 1.05–1.15) for register] and underestimate the lower prevalence of a contact with a GP [ORs 0.59 (95 % CI 0.51–0.70) and 0.41 (95 % CI 0.39–0.42) respectively] in Brussels compared to Flanders.

**Conclusion:**

Cautiousness is needed to interpret self-reported use of health care, especially for ambulatory care. Regional differences in self-reported health care use may be influenced by regional differences in the validity of the self-reported information.

**Electronic supplementary material:**

The online version of this article (doi:10.1186/s12874-016-0198-z) contains supplementary material, which is available to authorized users.

## Background

Information on the use of health care is an essential component of a health information system. Although it is well acknowledged that medical records and administrative data provide the most complete source of information on health care [1], health interview surveys remain an important additional source. First of all, medical records and administrative data are not without problems or inaccuracies [2–4]. Moreover, in many countries administrative and health data are not available in a format that allows producing nationwide information on the use of health care for all population groups. In contrast, survey data provide comprehensive information on a variety of services, and yet, are relatively inexpensive to obtain [5].

The collection of self-reported information on the use of health care is particularly useful for international comparisons. Within the European Union (EU), the need and demand for comparable comprehensive health data and health information has been well recognised [6]. In the past two decades substantial progress has been made towards the development of a permanent health monitoring and reporting system at the EU level [7]. One of the elements in this system is the European Health Interview Survey (EHIS), which includes also a module on use of health care (see Additional file 1). A European Commission regulation [8] guarantees that data obtained through this instrument are presently collected in all EU member states.

The collection of information on the use of health care via health interview surveys has also limitations. Two distinct types of bias may distort the results that are obtained through a population based survey: 1) a non-response or selection bias, if people who participate in the survey have a different consumption pattern than those who do not participate, and 2) a reporting bias, due to memory effects or misclassification by the respondents. Those biases may not only affect the validity of the estimates themselves, but also lead to an incorrect assessment of differences in the use of health care across population groups, e.g. regional differences.

A large body of literature has already investigated the validity of self-reported use of health care. However, many studies focused on specific population groups, were not representative and/or had small sample sizes [3, 9–11]. Some studies were based on national health surveys, but looked at other indicators than the ones used in the EHIS [12, 13]. None of the studies investigated concomitantly the selection and reporting bias or assessed if bias affected the outcome of regional differences in self-reported health care utilization.

In the present study, administrative data from the Belgian compulsory health insurance (BCHI) are used to investigate the validity of self-reported survey information on health care use from the Belgian Health Interview Survey (BHIS) 2008 and to explore to which extent the aforementioned selection bias and reporting bias affect the assessment of regional differences in health care use. Belgium is a country with 3 regions with quite different cultural, socio-economic and morphological characteristics: Flanders, the northern part in which people speak Dutch and which is the wealthiest part of the country, Wallonia, the southern region in which people speak French, and the Brussels Capital Region, which is an exclusively urban region with a large community of migrants and expatriates. The inclusion of EHIS questions in the Belgian national health survey in 2008 enables to explore the validity of regional differences in self-reported use of health care in Belgium for indicators that will be available during the coming years in all EU member states.

## Methods

### Data

The first dataset included the participants of the BHIS 2008. This survey was conducted between May 2008 and July 2009 among a representative sample of Belgian residents. A detailed description of the study design and sampling methods can be found elsewhere [14]. The response rate of the survey at household level, defined as the number of contacted households that participated in the survey, was 57 %. In total 11,253 people participated in the survey. If the selected person was not able to answer him/herself, a proxy interview was allowed. The study population was restricted to the population aged 15 years and over, resulting in a dataset of 9,651 individuals (BHIS total).

These data were linked by means of a unique identifier (the national number) with data from the BCHI. For the linkage an authorization was obtained from the Belgian Commission for the Protection of Privacy. For 424 individuals (4.4 % of total), the linkage was not possible; those people were consequently excluded from the study. In Belgium the health insurance is compulsory and covers more than 99 % of the population. However, for people working as an independent professional and their dependents (about 10 % of the population), complete coverage was not compulsory before 1 January 2008. For this reason no exhaustive information on reimbursed health care in the year preceding the interview was available for people with an independent profession (and their dependents) who participated in the survey before 1 January 2009 (*n* = 358). Also these people were removed from the dataset, resulting in a final sample of 8,869 individuals (BHIS linked), consisting of 91.9 % of the initial sample.

The second data set involved a large, unbiased random sample of the BCHI; the sample contains a population which is followed over time. Drop outs due to death and emigration are yearly replaced through a random procedure. A legal framework exists to use these data for policy and research purposes. The database used for this study consisted of the BCHI sample of the people aged 15 years and older for the year 2008 (*N* = 224,903).

Theoretically it was possible that a person was included in both the BHIS and the BCHI sample. For privacy reasons it was not possible to check this. However, as the probability of such an event was extremely low (roughly 1/40,000), it was assumed that both samples can be considered as mutually independent.

### Measures

A first set of measures was survey-based and thus only available in the BHIS. Outcome measures were at least one self-reported contact with a health care service or provider during the past 12 months and, for a contact with a dentist, also the past 6 months. Potential determinants that were explored were gender, age, region of residence, health status, measured through the presence of a chronic disease, illness or handicap, country of birth, education, household type, equivalent household income [15] and information on the person who answered the question (either the selected person him/herself or a proxy respondent).

A second set of measures was register-based and available both in the linked BHIS and the BCHI sample. The way in which the register-based outcome indicators were created, differed slightly by the source. In the linked BHIS the outcome was the prevalence of respondents with at least one registered contact with a health care facility in the 12 months (and for the dentist also in the 6 months) prior to the date of the interview. In the BCHI sample this was the prevalence of people with at least one contact with a health facility during the calendar year 2008. For a contact with the dentist in 6 months, the reference period was a random period of 6 months during the calendar year 2008. Age, gender and region were also available as register-based information. Other register-based measures for which it was estimated that they may have an impact on the validity of self-reported health care were 1) the type of insurance coverage, which essentially indicates whether the person was self-employed (or depended on a self-employed person) or not, 2) whether the person was eligible for preferential reimbursement, which corresponds with a vulnerable socio-economic situation, and 3) the natural logarithm of health care expenses in the year after the survey. The latter is a proxy for the intensity of the use of health care. The health care expenses in the year ‘after’ the survey were used, rather than those in the year ‘before’ the survey, to avoid any interference with the outcome indicators. Natural logarithmic transformation was performed to account for the skewedness of the health cost data. One euro was added to all costs to enable a logarithmic transformation for people who had not incurred any health costs (only 5.7 % of the population).

A final set of outcome measures was based on combined survey-based and register-based information in the linked BHIS. For each type of health care service, variables were constructed indicating whether the BHIS confirmed the information from the BCHI (accurate reporting), generated a false positive result (overreporting), or a false negative result (underreporting).

### Analyses

All outcome measures in the study were analysed at the individual level. In a first step, register-based information in the BCHI sample and the linked BHIS was used to assess the selection bias. Next, self-reported and register-based information from the linked BHIS was used to assess the reporting bias. A last set of analyses focused on the exploration of regional differences in the validity of self-reported health care and other determinants.

For each outcome indicator, the prevalence of a contact during the reference period was calculated in several ways: register-based use of health care in the BCHI sample and the linked BHIS, self-reported use of health care in the linked BHIS and the total BHIS. The reporting bias was assessed by calculating in the linked BHIS the proportions of actual agreement between register-based and self-reported use of health care use, overreporting (false positives) and underreporting (false negatives). Concordance was assessed with Cohen’s kappa statistic, which allows the assessment of “agreement beyond chance”. As kappa is affected by the prevalence of the finding under consideration [16, 17] and for rare findings very low values of kappa may not necessarily reflect low rates of overall agreement [18], also the positive and negative predictive values were calculated, as suggested by Cicchetti and Feinstein [19].

Regional variations in the validity of self-reported health care use were first explored via a stratified approach. For each region and each indicator absolute differences in the prevalence of a contact with a health care service/provider and a 95 % confidence interval were calculated according to different scenarios. In a further step, regional differences in the use of health care were investigated with logistic regression models. Age and sex adjusted odds ratios were calculated separately for register-based outcomes in the BCHI sample and the linked BHIS and self-reported outcomes in the total BHIS. By combining the BCHI sample and the total BHIS sample in one dataset, with the source as extra variable and the inclusion of an interaction between source and region in the model, it was possible to test if the observed regional differences varied significantly between self-reported data from the BHIS and register-based data from the BCHI sample.

Regional differences in over- and underreporting were studied by computing relative risk ratios (RRRs) from a multinomial logistic regression model. RRRs refer to the exponentiated coefficients from the model and have to be interpreted as the ratios of two relative risks. E.g., in a multinomial logistic regression model with over-, under- and accurate reporting as dependent variable and region as independent variable, the RRR for overreporting is the ratio between the relative risk of overreporting compared to accurate reporting in region X and the same relative risk in the reference region. In a first model, adjustment was made for age and sex only; in a second model a wide range of other potential confounders were included. Finally, the impact of those other potential confounders on inaccurate reporting, defined as either over- or underreporting, was assessed through a binomial logistic regression.

Analyses were performed with Stata 13.0 [20]. Survey data were analysed taking into account the multistage stratified clustered sampling design of the health survey. In the combined dataset population units from the BCHI sample were given a weight of 1.

## Results

### Validity of self-reported use of health care

Table 1 presents the distribution by age, gender and region of both the BHIS and the BCHI sample.

**Table 1:** Basic characteristics of Belgian Health Interview Survey (BHIS) and Belgian Compulsory Health Insurance (BCHI) samples, 2008

	BHIS		BCHI	
	Weighted % ^a^	*n*	%	*n*
Gender				
Men	48.12	4,417	48.69	109,497
Women	51.88	5,234	51.31	115,406
Age group				
15–24 years	14.27	1,209	14.41	32,407
25–34 years	15.30	1,340	15.31	34,427
35–44 years	17.92	1,503	17.11	38,482
45–54 years	17.88	1,403	17.20	38,677
55–64 years	14.09	1,337	14.37	32,324
65–75 years	10.32	879	10.41	23,408
75+ years	10.22	1,980	11.20	25,178
Region ^b^				
Flanders	58.20	3,411	58,81	129,902
Brussels	10.48	2,831	9,25	20,441
Wallonia	31.32	3,409	31,93	70,523
Total	100.00	9,651	100.00	224,903

^a^ Percentages are adjusted to the composition of the Belgian population in terms of age group, gender, province and household size through the use of post stratification weights

^b^ In the BCHI data, information on Region is absent for 4,037 people (1.79 % of sample)

In Table 2, the comparison of column 1 and 2 assesses the selection bias on the estimates of the self-reported health care use (based on the EHIS questions). For all register-based indicators but one, the linked BHIS yields slightly higher prevalences than the BCHI sample. The relative differences range from 4.2 % to 10.7 %. A lower estimate in the linked BHIS is obtained for an inpatient hospitalisation in the past 12 months, but also here the relative difference is rather small (- 7.6 %).

**Table 2:** Utilization of various types of health care, by source and assessment method, Belgium, 2008 (population aged 15 years and over)

	BCHI sample ^a^ (*n* = 224,903)		BHIS-linked ^b^ (*n* = 8,869)		BHIS-linked ^b^ (*n* =8,869)		BHIS-total ^c^ (*n* = 9,651)	
	Register-based information				Self-reported information			
	%	95 % CI	%	95 % CI	%	95 % CI	%	95 % CI
GP contact < 12 m	79.4	(79.2–79.5) ^d^	82.7	(81.5–83.8) ^f^	80.4	(79.0–81.4) ^f^	79.5	(78.3–80.8)
Specialist contact < 12 m	60.0	(59.8–60.2)	65.0	(63.5–66.4) ^f^	50.5	(48.9–52.1) ^f^	49.5	(48.0–51.0)
Dentist contact < 12 m	45.0	(44.8–45.2)	48.6	(46.9–50.2) ^f^	58.3	(56.5–60.0) ^f^	58.7	(57.1–60.3)
Dentist contact < 6 m	30.1	(29.9–30.3)	31.7	(30.2–33.3) ^f^	32.4	(30.8–34.0) ^f^	32.5	(31.0–34.0)
Physiotherapist contact < 12 m	15.9	(15.7–16.0) ^e^	17.6	(16.4–18.8) ^f^	17.3	(16.2–18.5) ^f^	17.1	(16.1–18.2)
Inpatient hospitalisation < 12 m	11.9	(11.7–12.0)	11.0	(10.1–12.0)	12.0	(11.0–13.0)	11.8	(10.8–12.7)
Day patient hospitalisation < 12 m	13.0	(12.8–13.1)	13.9	(12.9–14.9)	7.8	(7.1–8.6)	7.5	(6.8–8.3)

<12 m: the exact meaning differs slightly by source; for the BCHI sample: during the 12 months of 2008; for the BHIS sample: during the 12 months prior to the date of the interview

< 6 m: the exact meaning differs slightly by source; for the BCHI sample: during a random period of 6 months in 2008; for the BHIS sample: during the 6 months prior to the date of the interview

^a^ BCHI : Belgian Compulsory Health Insurance

^b^ BHIS-linked: Belgian Health Interview Survey Sample for which linkage was possible

^c^ BHIS-total: Total Belgian Health Interview Survey Sample

^d^ excluding 1.4 % of the population using a forfait system for reimbursement of GP services

^e^ excluding 1.2 % of the population using a forfait system for reimbursement of physiotherapist

^f^ excluding 5.9 % of sample not reimbursed for ambulatory care via the compulsory scheme during the whole study period

The comparison of self-reported (column 3) and register-based (column 2) outcomes in the linked BHIS allows to assess the reporting bias; it appears that for a contact with a GP and a physiotherapist in the past 12 months, a contact with a dentist in the past 6 months and an inpatient hospitalisation in the past 12 months, the estimates are more or less similar. Relative differences range from -9.1 % to 2.8 %. However, self-reporting strongly overestimates the prevalence of a contact with the dentist in the past 12 months (relative difference of 20.0 %) and strongly underestimates the prevalence of a contact with a specialist (relative difference of -22,3 %) and a day patient hospitalisation (relative difference of -43.9 %) during the same period.

A comparison of column 3 and 4 in Table 2 shows that there is hardly any difference between self-reported estimates in the linked BHIS and the total BHIS.

For a contact with a dentist, a physiotherapist and an inpatient hospitalisation, the overall concordance between reported and register-based use of care is fair to good, with kappas varying from 0.59 to 0.75 (Table 3). Regarding a GP contact, a specialist contact and a day patient hospitalisation, the agreement is moderate (kappa between 0.43 and 0.49). Most striking is the high percentage of false positives (or overreporting) for a contact with a dentist in the past 12 months (14.6 %) and the high percentage of false negatives (or underreporting) for a contact with the specialist in the past 12 months (19.5 %). The latter observation was further explored looking at the type of specialist (data not shown). After adjusting for contacts with other types of specialists, a false negative self-report with a specialist is significantly associated with a contact with a stomatologist (OR 6.15; 95 % CI 2.77–13.63), an ophthalmologist (OR 4.03; 95 % CI 3.00–5.42), a dermatologist (OR 3.30; 95 % CI 2.32–4.68), a psychiatrist (OR 2.12; 95 % CI 1.10–4.08) and an orthopaedic surgeon (OR 2.00; 95 % CI 1.38–2.90) among men, and with a contact with an ophthalmologist (OR 2.06; 95 % CI 1.63–2.61), a pneumologist (OR 1.91; 95 % CI 1.03–3.52), an ENT specialist (OR 1.81; 95 % CI 1.26–2.59) and a gynaecologist (OR 1.41; 95 % CI 1.11–1.78) among women.

**Table 3:** Concordance between self-reported and registered-based health care utilization (using the latter as reference), Belgian Health Interview Survey 2008 (population aged 15 years and over)

	Accurate reporting		Overreporting ^a^		Underreporting ^b^		Agreement		PPV ^c^		NPV ^d^	
	%	(95 % CI)	%	(95 % CI)	%	(95 % CI)	kappa	(95 % CI)	%	(95 % CI)	%	(95 % CI)
Contact GP < 12 m	84.8	(83.7–85.9)	5.9	(5.3–6.7)	9.2	(8.4–10.2)	0.47	(0.45–0.50)	91.5	(90.8–92.2)	53.6	(51.1–56.1)
Contact specialist < 12 m	75.6	(74.3–76.9)	4.9	(4.3–5.5)	19.5	(18.3–20.8)	0.49	(0.47–0.51)	89.2	(88.2–90.1)	58.7	(57.2–60.3)
Contact dentist < 12 m	80.3	(79.0–81.5)	14.6	(13.5–15.8)	5.1	(4.5–5.8)	0.59	(0.57–0.60)	71.6	(70.2–72.9)	87.7	(86.7–88.7)
Contact dentist < 6 m	85.2	(84.0–86.3)	7.7	(6.9–8.6)	7.1	(6.3–8.0)	0.65	(0.63–0.67)	74.3	(72.5–76.0)	90.2	(89.4–91.0)
Contact physiotherapist < 12 m	88.3	(87.3–89.3)	5.6	(5.0–6.3)	6	(5.3–6.9)	0.60	(0.58–0.62)	67.9	(65.5–70.2)	92.2	(91.5–92.8)
Inpatient hospitalisation < 12 m	95.2	(94.5–95.7)	2.9	(2.5–3.4)	1.9	(1.6–2.3)	0.75	(0.72–0.77)	75.7	(73.1–78.1)	97.2	(96.8–97.5)
Day patient hospitalisation < 12 m	89.7	(88.8–90.5)	2.1	(1.7–2.5)	8.2	(7.5–9.1)	0.43	(0.40–0.46)	69.7	(66.1–73.2)	90.5	(89.8–91.1)

< 12 m: in the 12 months prior to the date of the interview

< 6 m: in the 6 months prior to the date of the interview

^a^ Overreporting: self-reported contact, but no register-based contact

^b^ Underreporting: no self-reported contact, but register-based contact

^c^ PPV: positive predictive value

^d^ NPV: negative predictive value

The best agreement between reported and register-based use of health care is obtained for an inpatient hospitalisation in the past 12 months: 95.2 % accurate reporting, a kappa of 0.75, a positive predictive value of 75.7 % and a negative predictive value of 97.2 %.

### Validity of regional differences in self-reported use of health care

Table 4 shows how age and gender adjusted regional differences in the prevalence of a contact with a health care provider vary in function of the sample (BCHI sample versus BHIS) and the assessment method (register-based versus self-reported). The estimation that can be used as gold standard is the registered information on the use of health care in the BCHI. There is few variation in regional differences in register-based use of health care (column 1 and 2) between the two samples. On the other hand, the assessment of regional differences shows different results for self-reported health care in the BHIS than for register-based use of health care in the BCHI sample. For instance, self-reported survey data appear to underestimate the lower prevalence of a contact with a GP and overestimate the higher prevalence of a contact with the specialist in the Brussels’ Region.

**Table 4:** Odds ratios for use of health care in Brussels and Wallonia compared to Flanders, after adjustment for differences in age and gender, by source and assessment method, BHIS 2008

	Register-based use in BCHI sample ^a^ (*N* = 224,903)		Register-based use in BHIS-linked ^b^ (*N* = 8,869)			Self-reported use in BHIS-total ^c^ (*N* = 9,651)		
Contact with …	OR	(95 % CI)	OR	(95 % CI)	*P-*value interaction ^d^	OR	(95 % CI)	*P-*value interaction ^e^
GP < 12 m								
Flanders	1.00		1.00			1.00		
Brussels	0.41*	(0.39–0.42)	0.45*	(0.37–0.54)	0.21	0.59*	(0.50–0.70)	< 0.001
Wallonia	0.77*	(0.75–0.79)	0.79*	(0.65–0.95)	0.84	0.85	(0.72–1.01)	0.28
Specialist < 12 m								
Flanders	1.00		1.00			1.00		
Brussels	1.08*	(1.05–1.12)	1.33*	(1.15–1.53)	0.01	1.51*	(1.33–1.72)	< 0.001
Wallonia	1.19*	(1.17–1.22)	1.36*	(1.18–1.57)	0.10	1.26*	(1.11–1.43)	0.46
Dentist < 12 m								
Flanders	1.00		1.00			1.00		
Brussels	0.77*	(0.74–0.79)	0.78*	(0.67–0.89)	0.81	0.88	(0.76–1.02)	0.02
Wallonia	0.72*	(0.70–0.73)	0.70*	(0.61–0.81)	0.79	0.74*	(0.64–0.86)	0.47
Dentist < 6 m								
Flanders	1.00		1.00			1.00		
Brussels	0.85*	(0.83–0.88)	0.83*	(0.71–0.96)	0.65	0.91	(0.78–1.05)	0.42
Wallonia	0.77*	(0.76–0.79)	0.74*	(0.64–0.86)	0.56	0.73*	(0.63–0.85)	0.52
Physio < 12 m								
Flanders	1.00		1.00			1.00		
Brussels	0.90*	(0.86–0.94)	0.90	(0.76–1.07)	0.72	1.08	(0.92–1.27)	0.02
Wallonia	0.97*	(0.94–0.99)	0.86	(0.73–1.02)	0.16	0.92	(0.78–1.08)	0.53
Inpatient hospital < 12 m								
Flanders	1.00		1.00			1.00		
Brussels	0.92*	(0.88–0.97)	0.98	(0.80–1.20)	0.56	0.92	(0.76–1.11)	0.94
Wallonia	1.06	(1.03–1.09)	0.89	(0.73–1.08)	0.08	0.84	(0.70–1.01)	0.01
Day patient hospital < 12 m								
Flanders	1.00		1.00			1.00		
Brussels	0.94*	(0.90–0.98)	1.02	(0.86–1.22)	0.45	0.93	(0.74–1.16)	0.74
Wallonia	0.89*	(0.86–0.91)	0.89	(0.75–1.06)	0.95	0.79*	(0.63–0.99)	0.29

< 12 m: the exact definition differs slightly by source; for the BCHI sample: during the 12 months of 2008; for the BHIS sample: during the 12 months prior to the date of the interview

< 6 m: the exact definition differs slightly by source: for the BCHI sample: during a random period of 6 months in 2008; for the BHIS sample: during the 6 months prior to the date of the interview

* significant difference (*p* < 0.05)

^a^ BCHI : Belgian compulsory health insurance

^b^ BHIS-linked: Belgian Health Interview Survey Sample for which linkage was possible

^c^ BHIS-total: Total Belgian Health Interview Survey Sample

^d^ Interaction assessing if regional differences in register-based use are significantly different by source

^e^ Interaction assessing if regional differences in self-reported use of health care in BHIS are significantly different from those in register-based use of health care in BCHI sample

**Fig. 1:**
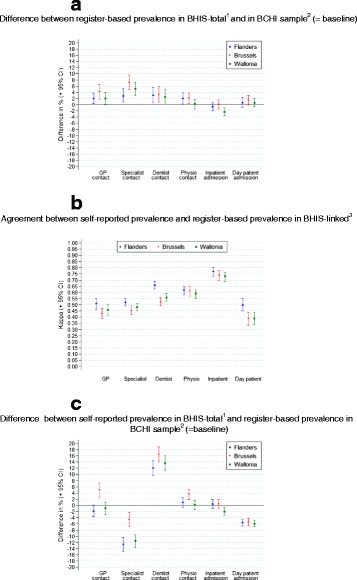
(**a**) Selection bias (**b**) Reporting bias (**c**) Selection and reporting bias, ^1^Total Belgian Health Interview Sample ^2^Sample Belgian compulsory health insurance ^3^Belgian Health Interview Survey Sample for which linkage was possible

This is confirmed in Fig. 1, which shows the difference between self-reported use of health care in the BHIS and register-based use of health care in the BCHI sample in the three Belgian regions, disentangling the selection and reporting bias. Figure 1c provides the combined effect of both biases. Regional differences in the validity of the estimate are most pronounced for a contact with a GP and a contact with a specialist. The difference in the prevalence of the population with a contact with the GP in the past 12 months between the self-reported estimate in the BHIS and the register-based estimate in the BCHI sample is smaller in Flanders (-1.8 %; 95 % CI–3.6 %;0.1 %) and Wallonia (-0.9 %;95 % CI–3.0 %;1.1 %) than in the Brussels’ Region (5.0 %; 95 % CI 2.7 %;7.2 %). For a contact with a specialist, the difference is bigger in Flanders (-12.6 %; 95 % CI–14.8 %;–10.3 %) and Wallonia (-11.5 %; 95 % CI -13.7 %;–9.4 %) than in Brussels (-4.5 %; 95 % CI–6.8 %;–2.2 %).

Table 5 provides information on regional differences of over- and underreporting a contact with a health service. In some cases, over- and underreporting level out, as is for instance the case for differences between Flanders and Wallonia in the prevalence of a contact with the dentist in the past 12 months. In Wallonia significantly more respondents inaccurately report a contact with the dentist in the past 12 months than in Flanders (OR 1.42; 95 % CI 1.16–1.76). At the same time significantly more respondents inaccurately report not to have had a contact with the dentist in Wallonia, compared to Flanders (OR 1.79; 95 % CI 1.31–2.45). As a result the odds ratio of a contact with a dentist in the past 12 months in Wallonia compared to Flanders is quite similar for self-reported information (OR 0.74; 95 % CI 0.64–0.86) as for register-based information (OR 0.70; 95 % CI 0.61–0.81) (Table 4).

**Table 5:** Regional differences in overreporting ^a^ and underreporting ^b^. Results of multinomial logistic regression, Belgian Health Interview Survey 2008 (population aged 15 years and over)

	Adjusted for age and gender				Also adjusted for other characteristics ^c^			
	Overreporting		Underreporting		Overreporting		Underreporting	
	RRR ^d^	(95 % CI)	RRR ^d^	(95 % CI)	RRR ^d^	(95 % CI)	RRR ^d^	(95 % CI)
Contact GP < 12 m								
Flanders	1.00		1.00		1.00		1.00	
Brussels	2.43*	(1.83–3.23)	1.38*	(1.07–1.79)	1.93*	(1.37–2.73)	1.52*	(1.12–2.07)
Wallonia	1.49*	(1.10–2.01)	1.30	(0.99–1.70)	1.55*	(1.14–2.11)	1.29	(0.97–1.70)
Contact specialist < 12 m								
Flanders	1.00		1.00		1.00		1.00	
Brussels	1.56*	(1.18–2.07)	1.00	(0.83–1.21)	1.32	(0.95–1.82)	1.10	(0.89–1.36)
Wallonia	1.13	(0.84–1.53)	1.16	(0.97–1.39)	1.14	(0.84–1.54)	1.16	(0.96–1.39)
Contact dentist < 12 m								
Flanders	1.00		1.00		1.00		1.00	
Brussels	1.54*	(1.25–1.90)	1.60*	(1.16–2.21)	1.46*	(1.16–1.85)	1.46*	(1.01–2.12)
Wallonia	1.42*	(1.16–1.76)	1.79*	(1.31–2.45)	1.45*	(1.18–1.79)	1.72*	(1.25–2.36)
Contact dentist < 6 m								
Flanders	1.00		1.00		1.00		1.00	
Brussels	1.45*	(1.11–1.88)	1.03	(0.77–1.39)	1.24	(0.93–1.68)	1.07	(0.79–1.46)
Wallonia	1.10	(0.84–1.44)	1.04	(0.78–1.39)	1.08	(0.83–1.42)	1.05	(0.79–1.40)
Contact physiotherapist < 12 m								
Flanders	1.00		1.00		1.00		1.00	
Brussels	1.13	(0.85–1.49)	0.70*	(0.51–0.97)	0.94	(0.69–1.29)	0.73	(0.51–1.06)
Wallonia	1.11	(0.83–1.47)	0.93	(0.69–1.27)	1.11	(0.84–1.47)	0.93	(0.68–1.28)
Inpatient hospitalisation < 12 m								
Flanders	1.00		1.00		1.00		1.00	
Brussels	0.97	(0.63–1.49)	1.43	(0.84–2.42)	0.73	(0.41–1.30)	1.50	(0.81–2.80)
Wallonia	0.96	(0.67–1.39)	1.39	(0.87–2.23)	0.86	(0.60–1.25)	1.44	(0.90–2.29)
Day patient hospitalisation < 12 m								
Flanders	1.00		1.00		1.00		1.00	
Brussels	1.09	(0.67–1.77)	1.25	(0.97–1.60)	0.74	(0.42–1.30)	1.43*	(1.09–1.86)
Wallonia	1.22	(0.78–1.91)	1.08	(0.84–1.38)	1.17	(0.75–1.81)	1.14	(0.90–1.46)

< 12 m : in the 12 months prior to the date of the interview

< 6 m : in the 6 months prior to the date of the interview

* significant difference (*p* < 0.05)

^a^ overreporting: self-reported contact, but no register-based contact

^b^ underreporting: no self-reported contact, but register-based contact

^c^ household type, education, income, type of insurance coverage, reimbursement schedule, country of birth, chronic disease, illness or handicap, proxy interview and health care

^d^ Relative Risk Ratio

Only if regional differences in overreporting are substantially different from regional differences in underreporting, as is for instance the case for a contact with a GP and a specialist in Brussels compared to Flanders, the association between use of health care and region yields different results for self-reported and register-based outcome indicators.

The logistic regression analyses indicate that, also after adjustment for a wide range of potential determinants, regional differences in over and/or underreporting in the use of health care are observed for self-reports of a contact with a GP, a contact with a dentist and a day patient hospitalisation.

### Determinants of inaccurate reporting

Table 6 provides information on the factors associated with inaccurate reporting. The association with region confirms the results in Table 5. Women tend to be less inaccurate in reporting a contact with the GP than men, but they are more inaccurate in reporting a contact with the specialist and the dentist. Higher age is associated with less inaccurate reporting of a contact with the GP and the dentist. Age differences in the accuracy to report a hospital admission do no show a consistent pattern. There appears to be no significant association between inaccurate reporting and socio-economic variables, such as education, income and being eligible for preferential reimbursement. The inaccuracy of reporting a contact is also related with chronic disease status and the volume of health care expenses, but the results differ by type of health care provider or service. A proxy interview yields significantly more inaccurate reporting for a contact with the GP and the dentist, but not for a contact with the other health services that are investigated.

**Table 6:** Determinants of inaccurate reporting ^a^ of at least one contact with a health care provider or hospital admission during a reference period. Results of binomial logistic regression (Belgian Health Interview Sample for which linkage was possible - population aged 15 years and over)

		*n*	GP < 12 m		Specialist <12 m		Dentist < 12 m		Dentist < 6 m		Physio < 12 m		Inpatient < 12 m		Day patient < 12 m	
			OR	(95 % CI)	OR	(95 % CI)	OR	(95 % CI)	OR	(95 % CI)	OR	(95 % CI)	OR	(95 % CI)	OR	(95 % CI)
Gender																
	Male	4,044	1.00		1.00		1.00		1.00		1.00		1.00		1.00	
	Female	4,825	0.75*	(0.62–0.90)	1.20*	(1.03–1.40)	1.02	(0.87–1.18)	1.19*	(1.00–1.41)	1.14	(0.92–1.41)	0.85	(0.62–1.15)	0.93	(0.75–1.15)
Age																
	15–24 yrs	1,092	1.00		1.00		1.00		1.00		1.00		1.00		1.00	
	25–34 yrs	1,187	1.17	(0.84–1.63)	0.88	(0.66–1.17)	0.67*	(0.50–0.88)	0.84	(0.60–1.19)	1.20	(0.76–1.91)	2.68*	(1.41–5.10)	0.77	(0.50–1.19)
	35–44 yrs	1,340	1.04	(0.77–1.39)	1.01	(0.78–1.30)	0.67*	(0.51–0.88)	0.88	(0.63–1.22)	1.05	(0.69–1.61)	1.28	(0.69–2.37)	0.85	(0.57–1.27)
	45–54 yrs	1,278	0.87	(0.63–1.19)	0.92	(0.70–1.19)	0.80	(0.61–1.05)	0.84	(0.60–1.17)	0.84	(0.54–1.32)	1.88*	(1.01–3.53)	0.76	(0.51–1.14)
	55–64 yrs	1,256	0.66*	(0.45–0.95)	0.92	(0.68–1.23)	0.59*	(0.43–0.82)	0.64*	(0.44–0.94)	0.89	(0.57–1.41)	1.62	(0.84–3.13)	0.53*	(0.34–0.83)
	65–74 yrs	829	0.46*	(0.28–0.74)	0.72	(0.51–1.02)	0.59*	(0.41–0.86)	0.52*	(0.33–0.82)	1.00	(0.60–1.66)	1.97	(0.94–4.13)	0.85	(0.53–1.35)
	75 yrs	1,887	0.43*	(0.27–0.68)	1.28	(0.93–1.77)	0.52*	(0.36–0.75)	0.34*	(0.22–0.53)	1.00	(0.62–1.61)	1.94	(0.97–3.88)	0.57*	(0.37–0.89)
Household type																
	Single	2,451	1.00		1.00		1.00		1.00		1.00		1.00		1.00	
	One parent with child (ren)	732	1.16	(0.80–1.69)	1.24	(0.89–1.72)	1.06	(0.75–1.48)	1.04	(0.72–1.50)	1.40	(0.91–2.14)	1.86*	(1.06–3.23)	1.15	(0.74–1.78)
	Couple with child (ren)	2,130	1.02	(0.75–1.39)	1.11	(0.90–1.37)	1.05	(0.82–1.34)	0.84	(0.63–1.13)	1.11	(0.83–1.48)	0.94	(0.64–1.38)	1.07	(0.80–1.45)
	Couple without child (ren)	2,698	1.18	(0.90–1.54)	1.21	(0.96–1.51)	0.98	(0.77–1.25)	1.01	(0.77–1.33)	1.31	(0.96–1.79)	1.49	(0.98–2.29)	1.02	(0.74–1.40)
	Other or unknown	858	1.66*	(1.18–2.35)	1.11	(0.83–1.48)	1.10	(0.78–1.54)	0.88	(0.61–1.27)	0.78	(0.51–1.20)	0.93	(0.54–1.60)	1.05	(0.70–1.59)
Region																
	Flanders	3,259	1.00		1.00		1.00		1.00		1.00		1.00		1.00	
	Brussels	2,464	1.74*	(1.37–2.21)	1.16	(0.96–1.40)	1.45*	(1.18–1.79)	1.17	(0.94–1.45)	0.84	(0.66–1.08)	0.95	(0.61–1.49)	1.24	(0.97–1.59)
	Wallonia	3,146	1.41*	(1.14–1.75)	1.15	(0.98–1.36)	1.51*	(1.26–1.81)	1.07	(0.87–1.31)	1.01	(0.81–1.26)	1.05	(0.78–1.41)	1.15	(0.92–1.43)
Educational level																
	No or primary	1,362	1.01	(0.69–1.48)	1.19	(0.92–1.55)	1.02	(0.76–1.37)	0.81	(0.56–1.16)	1.05	(0.73–1.49)	1.56	(0.97–2.51)	1.21	(0.85–1.72)
	Lower secondary	1,459	1.23	(0.90–1.68)	1.07	(0.81–1.41)	1.00	(0.77–1.32)	1.03	(0.75–1.40)	0.94	(0.67–1.33)	1.28	(0.80–2.06)	0.87	(0.61–1.22)
	Higher secondary	2,667	1.11	(0.88–1.43)	1.14	(0.94–1.39)	1.02	(0.83–1.26)	0.91	(0.72–1.16)	0.96	(0.74–1.26)	1.25	(0.87–1.79)	1.01	(0.77–1.32)
	Higher	3,111	1.00		1.00		1.00		1.00		1.00		1.00		1.00	
Equivalent income																
	Quintile 1	1,594	1.33	(0.94–1.88)	1.09	(0.82–1.45)	0.87	(0.64–1.18)	0.81	(0.60–1.14)	1.09	(0.72–1.65)	1.26	(0.73–2.17)	0.70	(0.48–1.01)
	Quintile 2	1,532	0.88	(0.64–1.20)	1.10	(0.84–1.45)	0.95	(0.72–1.26)	0.92	(0.66–1.29)	0.87	(0.62–1.22)	1.47	(0.88–2.47)	0.72*	(0.52–1.00)
	Quintile 3	1,409	0.89	(0.65–1.21)	1.20	(0.94–1.54)	0.92	(0.70–1.20)	1.12	(0.82–1.52)	1.00	(0.72–1.39)	1.01	(0.62–1.65)	0.80	(0.56–1.15)
	Quintile 4	1,363	0.95	(0.71–1.26)	1.11	(0.87–1.42)	0.91	(0.69–1.20)	0.89	(0.68–1.19)	1.11	(0.81–1.53)	1.40	(0.87–2.25)	0.85	(0.60–1.21)
	Quintile 5	1,493	1.00		1.00		1.00		1.00		1.00		1.00		1.00	
Type of insurance coverage																
	General scheme	8,023	1.00		1.00		1.00		1.00		1.00		1.00		1.00	
	Scheme for independents	822	1.73*	(1.27–2.36)	1.13	(0.86–1.49)	1.51*	(1.12–2.02)	1.53*	(1.11–2.11)	1.25	(0.87–1.80)	0.52*	(0.30–0.92)	1.31	(0.83–2.06)
Eligible for preferential reimbursement																
	No	7,095	1.00		1.00		1.00		1.00		1.00		1.00		1.00	
	Yes	1,750	0.77	(0.55–1.08)	0.95	(0.75–1.21)	0.88	(0.67–1.14)	1.27	(0.94–1.72)	0.92	(0.67–1.26)	1.10	(0.75–1.59)	0.92	(0.68–1.24)
Country of birth																
	Belgium	7,388	1.00		1.00		1.00		1.00		1.00		1.00		1.00	
	other European country	778	1.01	(0.72–1.42)	0.91	(0.67–1.22)	1.13	(0.84–1.53)	1.04	(0.74–1.46)	1.19	(0.97–1.81)	1.04	(0.65–1.66)	0.90	(0.62–1.32)
	outside Europe	698	1.41	(0.98–2.02)	1.00	(0.74–1.37)	1.31	(0.97–1.78)	1.14	(0.80–1.61)	1.12	(0.77–1.63)	1.71	(0.79–3.69)	1.10	(0.73–1.68)
Chronic disease, illness or handicap																
	No	3,255	1.00		1.00		1.00		1.00		1.00		1.00		1.00	
	Yes	5,583	2.00*	(1.58–2.54)	1.60*	(1.33–1.93)	1.21*	(1.01–1.45)	1.20	(0.95–1.51)	0.74*	(0.59–0.93)	0.88	(0.63–1.22)	0.62*	(0.48–0.79)
Proxy interview																
	No	7,768	1.00		1.00		1.00		1.00		1.00		1.00		1.00	
	Yes	1,095	1.55*	(1.14–2.15)	1.25	(0.95–1.65)	1.48*	(1.13–1.94)	1.40*	(1.03–1.89)	1.16	(0.82–1.63)	0.64	(0.37–1.11)	0.95	(0.64–1.40)
Health care expenses in year after survey (log)		8,869	0.89*	(0.85–0.93)	0.96*	(0.92–1.00)	0.97	(0.93–1.01)	1.05	(1.00–1.10)	1.32*	(1.23–1.42)	1.40*	(1.23–1.59)	1.46*	(1.34–1.59)

< 12 m : in the 12 months prior to the date of the interview

< 6 m : in the 6 months prior to the date of the interview

* significant difference (*p* < 0.05)

^a^ either overreporting (self–reported contact, but no register-based contact) or underreporting (no self-reported contact, but register-based contact)

## Discussion

The present study explored the validity of self-reported use of health care in a national health survey, focusing especially on regional differences. The results indicate that compared to administrative data, self-reports in a health survey yield good estimates for the prevalence of a contact with a GP and a physiotherapist and an inpatient hospitalisation; on the other hand, they tend to underestimate the prevalence of a contact with a specialist or a day patient hospitalisation. Self-reporting underestimates the lower prevalence of a contact with a GP and overestimates the higher prevalence of a contact with a specialist in Brussels compared to Flanders.

Although the validity of self-reported health information in a health survey depends both on the selection and the reporting bias, most studies focus on the latter. Generally, validity studies comparing self-reported service use against administrative records show inconsistent findings. Some show a favourable level of congruency between data from the two sources, but others do not [5]. Factors that affect accuracy include age, health status and number of chronic health problems, cognitive abilities, recall time frame, type of utilization, utilization frequency, questionnaire design, mode of data collection and memory aids and probes [1, 2, 21].

An important strength of our study is that it allows assessing concomitantly the selection and reporting bias. The selection bias is rather limited and goes in the same direction for all type of health services, resulting in a slightly higher prevalence of a contact with a health service among survey participants than in the total population. This is in line with a study performed in the Netherlands which concluded that after correcting for differences in demographic variables, respondents and non-respondents differ in the utilization of several types of care, resulting in a small overestimation of utilization [22]. In another study it is reported that the link between health services use and survey non-response may go in different directions [23].

The reporting bias strongly depends on the type of health service that is investigated. It hardly affects the estimation of the year prevalence of a contact with a GP, a physiotherapist, and an inpatient hospitalisation. However, it results into a substantial underestimation of the year prevalence of a consultation with a specialist and day patient hospitalisation, and a serious overestimation of the year prevalence of a consultation with a dentist. Underreporting of a contact with the specialist occurs more among people who had a contact with specific types of specialists, such as a dermatologist, ophthalmologist or gynaecologist. Those specialists are in Belgium often consulted without a referral by a GP and outside a hospital setting. Perhaps this is the reason why there is a recall bias when a contact with a specialist needs to be reported.

Underreporting of a day patient hospitalisation may be due to underreporting of admissions for chemotherapy or kidney dialysis, which are common indications for a day patient hospitalisation, but because of their repetitive character, patients may not conceive this as a hospital admission.

The estimate of a self-reported contact with a dentist appears to be more correct for a reference period of 6 months than for 12 months. This could be due to memory effects. It is also related to the fact that when the reference period of 6 months is used, under- and overreporting level out, whereas for a reference period of 12 months, there is much more overreporting than underreporting. So even if the agreement is not very good, self-reported and register based estimates may be similar if overreporting and underreporting occur to the same extent. Overreporting a contact with the dentist may be related to social desirability, as people may not like to report that they have not consulted a dentist during the past 12 months.

Despite the fact that the study involved multiple testing, we did not apply a Bonferroni correction, as this is a very conservative strategy. Instead, we checked if statistically significant differences were associated with plausible patterns and tendencies.

The concomitant assessment of the selection and reporting bias allows identifying how both biases have an impact on the results. For most types of health services the direction of the biases are opposite to each other, but the reporting bias, predominates. Only for a contact with a dentist in the past 12 months both biases reinforce each towards an important overestimation.

In the present study we investigated the validity of the probability of a contact with a health care service. Many studies focus also on the validity of the quantity of self-reported contacts with a health service [4, 5, 9, 10, 12, 13, 21, 24–29]. This may yield different results. A study in Belgium [12], based on a linkage of data from the health interview survey 1997 with health insurance data, using the number of GP and specialist visits as outcome indicator, found no significant difference between mean self-reported and registered specialist utilization, which is in contradiction with our finding that the prevalence of a contact with a specialist is much lower if it is based on self-reported than on register-based information. This difference could be due the type of indicator (quantity of use versus probability of use), but also to the reference period which was not the same (2 months versus 12 months).

An important focus of this paper is to assess the validity of regional differences in use of health care based on self-reports. One of the core findings in the field of clinical practice variation is that geographical differences in health care utilization and spending are systematic (not just random noise), substantial, pervasive and persistent over time [30]. At the population level, geography has been identified as an important determinant of health care use and health expenditure [31, 32]. Therefore international comparisons on health care use are high on the agenda of international agencies such as the OECD, which produces on a regular basis reports and working papers comparing aspects of the use of health care in its member states [33, 34]. Differences between countries in the organisation of the health system make international comparisons based on administrative and health data difficult or not possible. Therefore geographical differences are often assessed with self-reported data [35, 36].

Previous research concluded that self-reporting offers a reasonably valid estimate of differences in utilization of health care between socioeconomic groups in the general population [37] and has no systematic impact on estimates of ethnic differences in health care utilization [38]. From the present study it appears that, even though the magnitude of the association is not always correctly assessed, self-reported data provide acceptable estimates on regional differences in the use of health care, either because there are no regional differences in over or underreporting, or because regional differences in underreporting and overreporting are of the same size and level out (as it is for instance the case for a contact with a dentist in the past 12 months). However, in that case the study of the determinants of the use of health care, based on self-reported use, will lack validity.

For five outcome indicators we observed a measurement error in the assessment of regional differences in the use of health care due to a reporting bias. Although this does not affect the direction of the associations, it has an impact on the magnitude of the associations, resulting in an over or underestimation of some regional differences. Selection bias only plays a minor role, albeit that the relative overestimation of a contact with a specialist in the Brussels’ Region is the result of both a selection bias and a reporting bias. Although inaccurate reporting is associated with a higher age, chronic disease, a proxy interview and the intensity of health services use, these characteristics do not explain why there are regional differences in over or underreporting. Gender, socio-economic status and country of birth have in our study a limited impact on the validity of self-reported health care use.

The present results are useful for the interpretation of geographical differences in the use of health care based on EHIS data obtained through the same survey instrument. The extrapolation of methodological conclusions from our research to cross-country comparisons in EU member states has of course limitations. In the Belgian context, the organisation of the health care, which has definitely an impact on the health care use, does not vary dramatically across regions, but health systems vary widely between European countries. Moreover, also other methodological aspects such as the place of a question in the questionnaire, mode of data collection, sampling method and recruitment, must be addressed to ensure harmonization in cross country comparisons [39, 40]. Obviously these aspects did not vary by region in the Belgian health survey. Therefore it is quite plausible that European cross-country comparisons in the use of health care will be more affected by validity problems than this is the case for regional differences in Belgium.

## Conclusions

The validity of self-reported use of health care, based on EHIS questions, varies by type of health service. Regional differences in the use of self-reported health care may be influenced by regional differences in the validity of the self-reported information.

This finding is important for cross-country comparisons between EU member states, based on the same instrument, especially as cross-country comparisons are more challenging than regional comparisons within one country.

Apart from EHIS, other large scale European surveys, like the European Union Statistics on Income and Living Conditions (EU-SILC) [41] and the Survey of Health, Ageing and Retirement in Europe (SHARE) [42] seek to obtain comparable health data across Europe. A critical reflection on the impact of both the selection and reporting bias on the validity of international comparisons based on survey data, remains important and should be included in the future research agenda.

## Abbreviations

EHIS, European health interview survey; BHIS, Belgian health interview survey; BCHI, Belgian compulsory health insurance; EU, European Union; GP, general practitioner; OECD, organisation for economic co-operation and development; EU-SILC, European Union statistics on income and living conditions; SHARE, survey of health, ageing and retirement in Europe; RRR, relative risk ratio

## Additional file

Additional file 1: European Health Care Module in the European Health Interview Survey. (DOCX 30 kb)
