# Supplementary material for: Regional differences in the validity of self-reported use of health care in Belgium: selection versus reporting bias
Source: BMC Med Res Methodol. 2016 Aug 16;16:98. doi: 10.1186/s12874-016-0198-z (PMC4986374; doi:10.1186/s12874-016-0198-z)
Supplement: Additional file 1: — European Health Care Module in the European Health Interview Survey. (DOCX 30 kb) [file 12874_2016_198_MOESM1_ESM.docx]

**European Health Care Module in the European Health Interview Survey**

The next set of questions is about time spent in hospital. All types of hospitals are included.

HO1. In the past 12 months have you been in hospital as an inpatient, that is overnight or longer? (Yes/No)

HO2. Thinking of all these occasions you have been an inpatient, how many nights in total did you spend in hospital?

HO3. In the past 12 months, have you been admitted to hospital as a day patient, that is admitted to hospital for diagnosis, treatment or other types of health care, but not required to remain overnight? (Yes/No)

HO4. In the past 12 months how many times have you been admitted to hospital as a day patient?

The next question is about visits to dentists, orthodontists or other dental care specialist.

AM1. When was the last time you visited a dentist or orthodontist on your own behalf (that is, not while only accompanying a child, spouse, etc.)? (Less than 6 months / 6 to less than 12 months / 12 months or longer / Never)

The next set of questions is about consultations with your general practitioner or family doctor. Please include visits to your doctor’s office as well as home visits and consultations by telephone.

AM2. When was the last time you consulted a GP (general practitioner) or family doctor on your own behalf? (Less than 12 months ago / 12 months ago or longer / Never)

AM3. During the past four weeks ending yesterday, how many times did you consult a GP (general practitioner) or family doctor on your own behalf?

Next questions are about consultations with medical or surgical specialists. Include visits to doctors as outpatient or emergency departments only, but do not include contacts while in hospital as an in-patient or day-patient.

AM4. When was the last time you consulted a medical or surgical specialist on your own behalf? (Less than 12 months ago / 12 months ago or longer / Never)

AM5. During the past four weeks, how many times did you consult a specialist on your own behalf?

AM6. In the past 12 months have you visited on your own behalf a…?

A. Physiotherapist or kinesitherapist (Yes/No)

B. Psychologist, psychotherapist or psychiatrist (Yes/No)

The next question is about home care services that cover a wide range of health and social services provided to people with health problems at their homes. These services comprise for example [home care service provided by a nurse or midwife, home help for the housework or for elderly people, meals on wheels or transport service]. Only services provided by professional health or social workers should be included.

AM7. In the past 12 months, have you yourself used or received any home care services? (Yes/No)
